# Coverage with evidence development for medicines with insufficient evidence of clinical benefit: experience from the Netherlands

**DOI:** 10.1017/S0266462325103267

**Published:** 2025-11-17

**Authors:** Jan-Willem Versteeg, Noraly Stam, Aukje K. Mantel-Teeuwisse, Lonneke Timmers, Wim Goettsch, Christine Leopold

**Affiliations:** 1 Division of Pharmacoepidemiology and Clinical Pharmacology, Utrecht Institute for Pharmaceutical Science, Utrecht University, Utrecht, The Netherlands; 2 Zorginstituut Nederland, Diemen, The Netherlands; 3 Erasmus School of Health Policy and Management, Erasmus Universiteit Rotterdam, Rotterdam, The Netherlands

**Keywords:** coverage with evidence development, life-cycle HTA, orphan medicinal products, conditional & exceptional market authorization, real world evidence

## Abstract

**Objectives:**

Since 2019 the Dutch National Healthcare Institute has operated a coverage with evidence development (CED) program for specific products with insufficient evidence of their clinical benefit: orphan medicinal products (OMPs), medicines with conditional marketing authorization (CMA), and medicines with marketing authorization under exceptional circumstances (AEC). The objective of this study is to give an overview of this program and reflect on learnings, challenges, and opportunities.

**Methods:**

This study is a narrative policy review of the Dutch CED program and describes the different phases and stakeholder involvement. Additionally, an overview of the medicines included in the CED program is presented and put in an international perspective.

**Results:**

The CED program consists of four phases: candidate prescreening, research protocol drafting, signing of process agreement and financial agreement, and controlled access. Stakeholders are involved intensively throughout the process. Since 2019, six medicines have entered the program. The program is used to fill different evidence gaps for various types of medicines and indications. In other countries, these medicines are often included in restricted reimbursement programs.

**Conclusions:**

The CED program is gathering clinical effectiveness data while providing patient access to OMPs, CMA, and AEC products. Important facilitating factors for the program were identified, including the involvement of all stakeholders, the only-in-research approach of data gathering, and the case-by-case evidence generation requirements and duration. Continuous evaluation is needed as the program does not yet include the expected number of medicines, and no conclusion can be drawn so far on the usefulness of the data collection.

## Introduction

To promote access to promising medicines with an incomplete evidence package, the European Medicines Agency (EMA) has several special processes and mechanisms in place. For orphan medicinal products (OMPs), medicines that target rare diseases, there is a special orphan designation that entitles health technology developers (HTDs) to protocol assistance and longer market exclusivity (1). Additionally, EMA provides alternatives to the standard marketing authorization (MA), such as the conditional MA (CMA) and authorization under exceptional circumstances (AEC). CMAs and AECs are granted for medicines that often target an unmet medical need (always for CMAs) but come to market with less comprehensive data (2). OMPs might also have less comprehensive data, as they often come to market with studies with small sample sizes, no control group, surrogate outcomes, and/or limited follow-up duration (3). Since the introduction of these special procedures, their uptake has ever increased, meaning that over time, more and more medicines have entered the market with limited evidence of their clinical benefits (3;4).

This leads to challenges in the subsequent health technology assessment (HTA) and the reimbursement decision-making process. Compared to medicines that have received standard MA, medicines that received CMA or AEC are prone to receive negative or restricted reimbursement advice because of insufficient evidence on their clinical benefits and duration of effects, uncertainties due to study design, and issues in economic modeling (e.g., uncertainties regarding model structure and assumptions and the cost-effectiveness estimate) (5–7). However, when there are no alternative treatments available, patient access to these promising medicines might be desired, and further data collection is therefore warranted.

HTA organizations and payers often use different approaches to cope with the uncertainties of these medicines. Most commonly used are simple financial agreements in which these uncertainties are discounted. However, it is also possible to implement more complex managed entry agreements (MEAs). Outcome-based MEAs (OB-MEA) are agreements in which the reimbursement of a medicine is linked to the performance of the treatment and might be better suited in cases with large uncertainties regarding clinical benefit (8;9). Coverage with evidence development (CED) is a form of OB-MEA where temporary reimbursement is combined with parallel targeted data collection to reduce clinical uncertainty (10;11). CED programs have been implemented before; reviews of earlier CED programs in the United States, Switzerland, and the Netherlands found that the use of these programs seems promising but is also less effective than it could be. Important challenges described in these earlier reviews were setting up data collection, the duration of the CED program, and the reversal of a coverage decision if the evidence turns out to be negative (12–14) .

To accommodate access for OMPs, CMAs, and AECs, which cannot offer sufficient evidence of clinical benefit, the Dutch National Health Care Institute (Zorginstituut Nederland, ZIN) was commissioned by the Ministry Of Health (MOH) to implement the conditional inclusion program in 2019; *‘voorwaardelijke toelating weesgeneesmiddelen, conditionals en exceptionals’* (15). In this CED program, selected medicines are reimbursed conditionally after price negotiation, while further data are being collected to bridge the case-specific evidence gap. The CED program has been in place for several years now, and multiple medicines have been included in the program. This study aims to give a transparent and complete overview of the Dutch CED program and all its procedures (including involved stakeholders and needed evidence) and how it fits into the standard Dutch HTA procedure. Additionally, the study gives an overview of the medicines included in the program and describes the recommendations on these medicines by other international HTA organizations. Finally, the study aims to reflect on the aspects of the CED program that are going well and the aspects of the CED program that need improvement.

## Methods

### Study description

**Figure 1.**
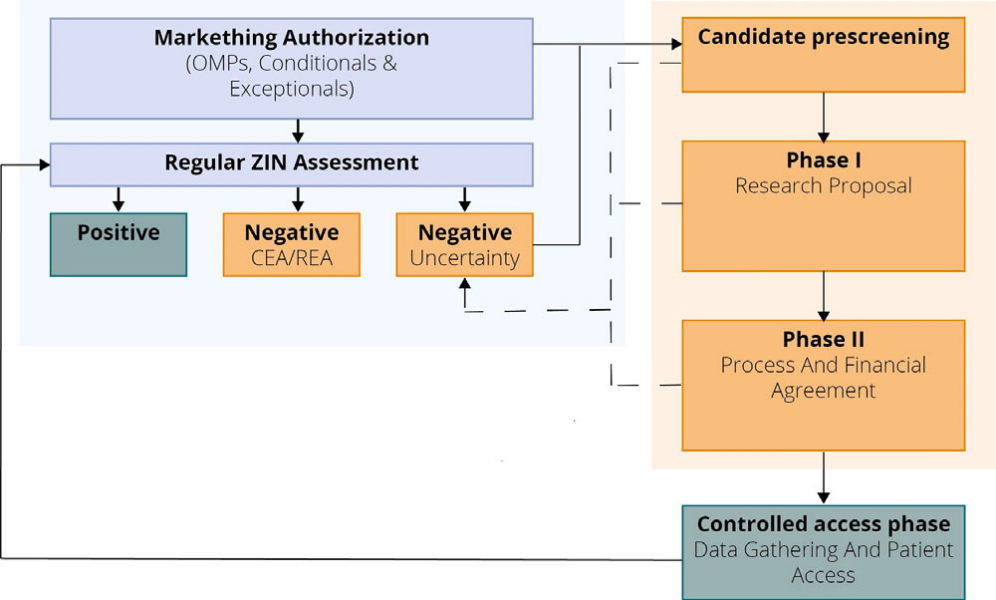
Schematic display of where the CED program fits in the regular ZIN assessment procedure. Abbreviations: CEA, cost-effectiveness analysis; OMPs, orphan medicinal products; REA, relative effectiveness assessment; ZIN, Zorginstituut Nederland.

**Figure 2.**
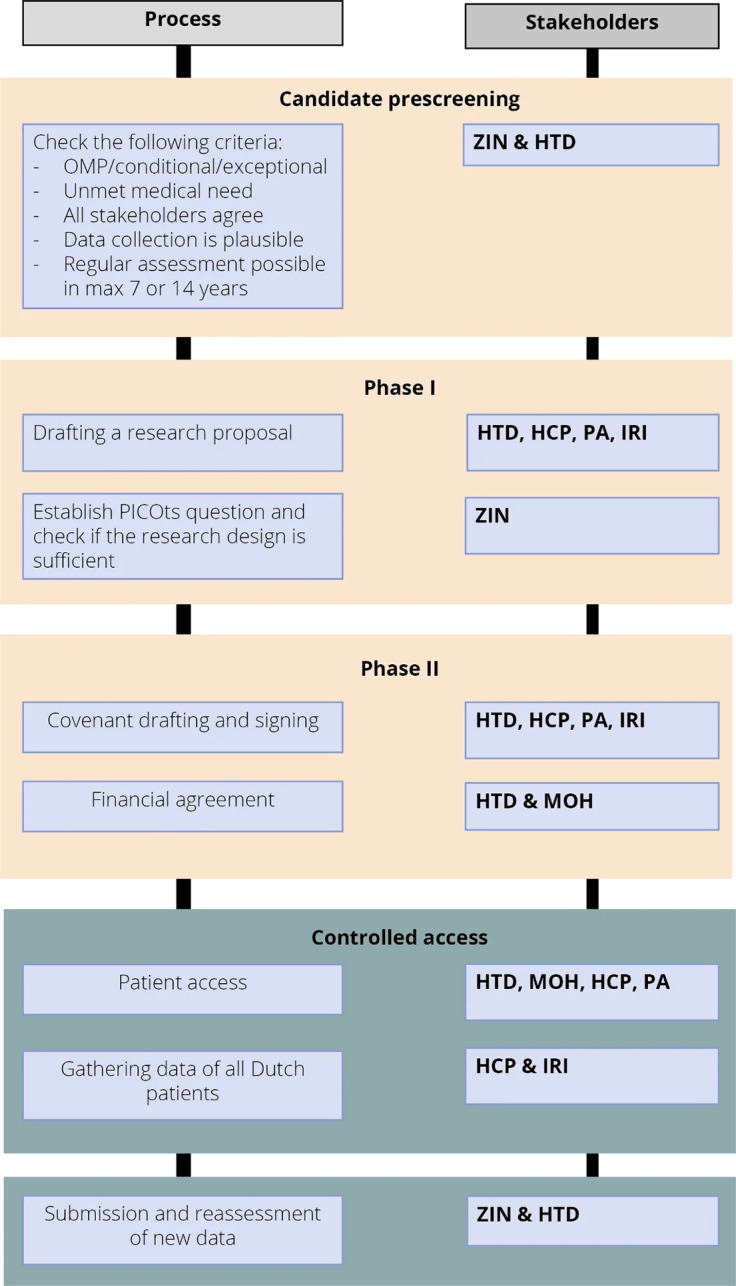
Detailed process description of the CED program with stakeholder involvement. Abbreviations: HCP, association of health care providers; HTD, health technology developer; IRI, Independent Research Institute; MOH, Ministry of Health; OMP, orphan medicinal product; PO, patient organization; ZIN, Dutch National Healthcare Institute.

This study is a narrative policy review of the Dutch CED program based on data collection, document analysis, and expert consultations. First, a description of the CED program implemented by ZIN, as commissioned by the MOH, is displayed in two flowcharts: one on the CED program within the Dutch reimbursement system (Figure 1) and the other on the process including stakeholder involvement (Figure 2). Second, characteristics of currently included drugs in the CED program are described. Finally, a comparison with other countries’ reimbursement recommendations for the included medicines is included.

### Data sources

Information used for the process description and case studies was obtained from publicly available ZIN documents, including, but not limited to, process descriptions, progress reports, evaluation reports, and HTA reports. Information for the international comparison was based on publicly published HTA reports by the included HTA organizations. Information regarding regulatory approval was obtained from European Public Assessment Reports (EPARs). In addition to the document analysis, experts from ZIN were consulted in all phases of this research: study design, result construction and interpretation, and manuscript drafting.

### Characteristics of products included in the CED program

The product basket includes all medicines that had been or are currently included or are in preparation for inclusion in April 2025. To describe the main characteristics of these products, data from EPARs and literature was extracted on medicine type (based on ATC code), therapeutic area, type of MA (standard MA, CMA, AEC), whether a medicine has an orphan designation, specific evidence requested by ZIN (and EMA for CMAs), method of data gathering for Dutch patients, the envisioned duration of the CED program, and (if applicable) actual duration. The scope and use of the CED program were subsequently analyzed based on the extracted data.

### International comparison

The selection of comparator countries was based on the public availability of HTA reports and languages known to the researchers (Dutch, English, German, and French). This led to including the following organizations: the National Institute for Health and Care Excellence (NICE; United Kingdom), Canada’s Drug Agency (CADTH; Canada), Institut für Qualität und Wirtschaftlichkeit im Gesundheitswesen/Der Gemeinsame Bundesausschuss (IQWIG/G-BA; Germany), and Haute Autorité de Santé (HAS; France). For the above-selected products, data on reimbursement recommendations, including access restrictions, were extracted from national HTA reports.

## Results

### Process of the CED program

In 2019, the CED program was implemented to ensure temporary and controlled access to promising medicines with market authorization that do not yet have sufficient evidence for their clinical benefit. During this time, additional information on effectiveness and appropriate use is collected, which is used to agree on a final reimbursement decision. Figure 1 describes the different phases of the CED program and how they fit into the regular ZIN assessment pathway. A regular ZIN assessment consists of a relative effectiveness assessment, a budget impact analysis, and a risk-based cost-effectiveness assessment (16;17).

In Figure 2, details of the CED process, including stakeholder involvement, are described. The process includes four stages: (i) candidate pre-screening, (ii) research proposal (called Phase I), (iii) process and financial agreement (called Phase II), and (iv) controlled patient access with evidence development. At the end of the controlled access stage, a reassessment is performed to make a final reimbursement decision.

#### Candidate pre-screening

Inclusion in the CED program can be initiated at two moments: either through an early submission by the HTD in case a regular reimbursement assessment is skipped (when it is already evident that the available evidence is insufficient for a positive assessment) or after a negative assessment (due to uncertainty about clinical benefit). ZIN then checks a medicine’s eligibility based on five criteria:

1. The medicinal product has been granted CMA or AEC by the EMA and/or is a designated OMP for the indication concerned. Off-label indications are not eligible for the CED program.
2. The medicinal product fulfills an unmet medical need, according to the EMA definition. This is defined as a condition for which no satisfactory method of diagnosis, prevention, or treatment exists, or, even if such a method exists, the medicinal product concerned will be of major therapeutic advantage to those affected.
3. In contrast to the regular reimbursement procedure, the application consists of multiple co-applicants. The HTD is the lead applicant of the conditional reimbursement dossier, and the co-applicants are an independent research institute, the association of the relevant health care providers, and the relevant patient organization.
4. It is plausible that the data collected through the proposed research proposal will provide sufficient evidence to decide whether the medicinal product in question warrants reimbursement.
5. It is plausible that the evidence needed can be collected within the period of temporary conditional reimbursement (max. 7 years or 14 years depending on the evidence gap and patient population),

Only if all criteria are met, the medicinal product can be eligible for inclusion in the CED program.

#### Phase I – Research proposal

After a medicinal product has been found eligible, the HTD submits a dossier containing a research proposal to determine the clinical benefit to ZIN. ZIN then assesses whether the results that are likely to follow from the research proposal will be of sufficient quality at the end of the conditional reimbursement period. ZIN examines the relevant patient population, the intervention, alternative treatment methods, relevant outcome measures, and the duration of the study (PICOt). Additional evidence can be either gathered in a (registry) study launched in the Netherlands or come from ongoing research required by the EMA in light of a CMA or other ongoing clinical trials. Data of all Dutch patients need to be collected during the conditional reimbursement period. So, when effectiveness data are expected from an ongoing international study, a supportive registry study in the Netherlands is required. As reimbursement is linked to data collection, the medicine is only reimbursed for patients participating in data collection.

#### Phase II – Process and financial agreement

If all selection criteria are met and the research proposal is of sufficient quality, ZIN advises the MOH that the medicinal product is a potential candidate for conditional reimbursement, and phase 2 can start. Phase 2 consists of 2 parallel processes: drafting up a process agreement (named “covenant”) and coming to a financial agreement. Only when all stakeholders sign the process agreement and a financial agreement is successfully negotiated, the trajectory can start.

#### Process agreement

To guarantee that all stakeholders agree to the proposed terms before the start of the conditional inclusion process, a detailed process agreement is signed between all stakeholders. The process agreement includes information about the proposed research, data collection, patient information, exit strategy, and a disinvestment plan. Moreover, possible future scenarios should be discussed; for example, what new medicines for the same indication are expected to be registered in the upcoming years, and what could this mean for the reimbursement? All stakeholders, as shown in Figure 2, sign this process agreement.

#### Financial agreement

The financial agreement is negotiated between the MOH and the HTD and includes the terms of the price negotiated for the medicinal product. The HTD will pay all other costs, e.g., the costs of data gathering as part of the CED program, for the full duration of the program.

#### Controlled access stage

When temporary conditional reimbursement starts, patients receive treatment with the included medicine, and data are gathered and analyzed following the research protocol and process agreement. The data are owned by the HTD or the health care professionals (HCPs), depending on whether data are collected in ongoing HTD-funded trials/registries or whether data is collected in an HCP-owned disease-specific registry. An independent research institute performs data analysis to ensure that appropriate analyses are performed. During the program, there are annual monitoring moments where progress and data quality and interim results are assessed, and, if necessary, the trajectory could be amended or terminated early.

### Current CED product basket

Table 1 shows the characteristics of the medicines currently included in the CED program. Since the implementation of the CED program in 2019, six products have been included or will probably be included soon as of April 2025: entrectinib (Rozlytrek®), larotrectinib (Vitrakvi®), ataluren (Translarna®), recombinant human parathyroid hormone 1–84 (rhPTH 1–84 – Natpar®), autologous CD34+ cells encoding the ARSA gene (CD34 – Libmeldy®), and teduglutide (Revestive®). Medicines that are or will be included in the CED program are pharmacologically diverse, with mechanisms of action ranging from kinase inhibitors (entrectinib and larotrectinib) to gene therapy (atidarsagene autotemcel) and hormone replacement medicines (rhPTH 1–84). Additionally, the medicines treat diverse indications ranging from tumor-agnostic oncology indications to rare inherited metabolic disorders such as metachromatic leukodystrophy. This is also reflected in the MA, with 4/6 medicines receiving CMA and 4/6 medicines receiving an orphan designation.

**Table 1.** A, B, and C: Background information of the six medicines that have, or will be, included in the coverage with evidence development program

INN	Ataluren	RhPTH 1–84	Atidarsagene autotemcel	Teduglutide	Entrectinib	Larotrectinib
Brand name	Translarna	Natpar	Libmeldy	Revestive	Rozyltrek	Vitrakvi
Date MA EMA	31–07–2014	24–4–2017	17–12–2020	30–8–2012	31–7–20	19–9–2019
Medicine Group	other drugs for disorders of the musculoskeletal system (m09a)	parathyroid hormones and analogs (h05a)	other nervous system drugs (no7)	other alimentary tract and metabolism products(a16a)	protein kinase inhibitors (l01e)	protein kinase inhibitors (l01e)
Disease area	muscular dystrophy, duchenne	hypoparathyroidism	leukodystrophy, metachromatic	malabsorption syndromes	cancer/carcinoma, non-small-cell lung	cancer/carcinoma, non-small-cell lung
Date prescreened	24–11–2020	12–2020	8–5–2023	21–09–2023	10–3–21	10–03–2021
Date final advice on conditional reimbursement	1–10–2021	1–10–2021	NA	11–02–2025	16–8–21	16–8–21
Date conditional reimbursement started	1–11–2021	1–11–2021	NA	01–04–2025	1–10–2021	1–10–2021
Duration envisioned	2 years 11 months (prolonged)	3 years 2 months	NA	5 years	3 years 2 months	3 years 2 months
Date final recommendation by ZIN	21–02–2024	21–02–2024	NA	NA	27–07–2023	27–07–2023
Number of days between conditional reimbursement and final recommendation by ZIN	NA	NA	NA	NA	664	664
Conditionally reimbursed indication	Patients of 2 years and older with Duchenne Muscular Dystrophy	Adjuvant treatment of adult patients with chronic Hypoparathyroidism uncontrollable by standard therapy	Treatment of children with early juvenile form of MLD with early clinical manifestation of the disease but still able to walk and no cognitive decline	Adult patients stable after a period of intestinal adaption following surgery. IBD must be the cause of the intestinal resection combined with intensive parenteral nutrition. Secondly patients from 6 months to 18 years who are dependent on parenteral nutrition for 30% of their intake	Patients with solid tumors that have an NTRK-gene fusion, a tumor agnostic indication	Patients with solid tumors that have an NTRK-gene fusion, a tumor agnostic indication
Orphan status	Yes	Yes	Yes	Yes	No	No
Marketing authorization route	CMA	CMA	Standard	Standard	CMA	CMA
MA evidence requested	The CHMP considered that the Applicant will complete a confirmatory randomized placebo-controlled Phase 3 study PTC124- GD–020-DMD (Study 020) with the 10, 10, 20 mg/kg/day dose in patients with nmDMD.	To further confirm the efficacy and safety of NATPAR in the treatment of patients with chronic hypoparathyroidism who cannot be adequately controlled with standard therapy alone, the MAH should conduct a randomized controlled trial comparing NATPAR to Standard of Care and to alternative dosing according to an agreed protocol.	NA	NA	1. A pooled analysis for an increased sample size of NTRK fusion-positive patients from the ongoing studies STARTRK–2, STARTRK-NG and any additional clinical trial conducted according to an agreed protocol. The MAH should submit the results of an interim safety and efficacy analysis of the NTRK efficacy-evaluable adult and pediatric patients, including adolescents that are available as per the integrated statistical analysis plan. 2. The MAH should submit the results from tumor genomic profiling by plasma and/or tissue when possible at baseline and progression together with clinical outcomes association per tumor histology for the patients from the updated pooled analysis.	1. the MAH should submit a pooled analysis for the increased sample size including the final report of the study LOXO-TRK–15002 (NAVIGATE). 2. The MAH should submit the final report of study LOXO-TRK–15003 (SCOUT) including 5-year follow-up data. 3. The MAH should submit an updated pop PK model based on additional PK sampling in patients aged 1 month to 6 years from study LOXO-TRK–15003 (SCOUT).
Evidence gap identified by ZIN	Effectiveness for so-called mid-range patients. ZIN wants at least 1. a clinically relevant effect in the primary outcome of trial 041 (effectiveness in mid-range patients) 2. More insights into how ataluren alters dystrophy production 3. A confirmation of decline for stable phase patients	The studied population did not match the population in the Dutch package question. In the registration trials, there was no specific condition set in the indication that patients had to be unable to control with standard therapy.	For the subgroup of early symptomatic patients, ZIN requests more data on efficacy as the pivotal study included too few patients	If there is an effect on quality of life in a specific sub-population of the indication	The current assessment framework is not ready for tumor-agnostic products	The current assessment framework is not ready for tumor-agnostic products
Evidence upon completion CED program	Likely to be terminated before completion	Terminated before completion	NA	NA	The assessment framework is updated in 2023. Single-arm trials and pooled analyses.	The assessment framework is updated in 2023. Single-arm trials and pooled analyses.
Dutch patients included in international ongoing clinical trials	Partly	No	No (no clinical trial)	No (no clinical trial)	Partly	Partly
Additional data collection	Duchenne-Ataluren register in the Netherlands (part of Dutch Dystrophinopathy Database DDD)	Additional Dutch research (NatPar monitor) since no Dutch patients could join the ongoing international studies.	International multicenter MLD registry study	Revestive monitor NL	Additional data gathering in the Netherlands on efficacy, Drug Access Protocol	Additional data gathering in the Netherlands on efficacy, Drug Access Protocol
Final recommendation by ZIN	Follow a negative CHMP decision and stop reimbursement. Likely no final reimbursement recommendation	Product taken off the market by manufacturer, no final reimbursement advice	NA	NA	REA; Positive – CEA; Not Applicable (senseless for tumor agnostic products) – Final recommendation: Positive reimbursement recommendation	REA; Positive – CEA; Not Applicable (senseless for tumor agnostic products) – Final recommendation: Positive reimbursement recommendation

Abbreviations: CHMP, Committee for Medicinal Products for Human Use; CMA, conditional marketing authorization; CEA, cost-effectiveness assessment; DMD, Duchenne Muscular Dystrophy; EMA, European Medicine Agency; INN, International Nonproprietary Name; MA, Marketing Authorization; MAH, marketing authorization holder; MLD, Metachromatic leukodystrophy; NA, not applicable; NTRK, neurotrophic tyrosine receptor kinase; REA, relative effectiveness assessment; ZIN, Zorginstituut Nederland.

### The case of larotrectinib and entrectinib

Larotrectinib and entrectinib are special cases, as they entered the CED program after it became apparent that ZIN’s current assessment framework could not assess their tumor-agnostic indications. A special extra phase was added to the program to achieve early access before entering the regular CED program. Larotrectinib and entrectinib did not go through the research proposal phase (phases I and II) of the CED program. During the controlled access phase, data regarding their clinical benefit was obtained through running clinical trials as part of the Dutch Drug Access Protocol (DAP) (18). This extra phase was introduced for the period in which ZIN developed its criteria for the assessment of tumor-agnostic drugs. Once this was done, both drugs were assessed using predominantly international data, resulting in regular reimbursement for both larotrectinib and entrectinib. The programs’ duration for larotrectinib and entrectinib was shorter than envisioned, running 22 months.

#### Overview of the regular program

Four medicines entered the original regular CED program: ataluren, rhPTH 1–84, teduglutide, and CD34 (with the last one still in phase II). Only one out of four medicines did not have a regular ZIN assessment before. RhPTH 1–84 entered the CED procedure after a pre-submission advice meeting with ZIN, which showed that the available data would be insufficient to answer the PICOt question.

The CED program is used to answer a variety of research gaps. For ataluren, more data was being gathered on a specific Duchenne patient subgroup. There was an international clinical trial still running for this population, as well as an international registry study. In the Netherlands, an additional registry was used to gather data from patients who could not be included in the international trials. For rhPTH 1–84, new data had to be gathered because the trial population did not properly reflect the Dutch population. There was an international trial running that gathered data, but no Dutch patients could be included anymore. Therefore, data from Dutch patients were gathered in a Dutch registry.

One of the four medicines that follow the regular CED program, ataluren, lost marketing authorization after four negative opinions from the Committee for Medicinal Products for Human Use (CHMP) between September 2023 and October 2024. The European Committee (EC) has taken up the CHMP’s advice to terminate ataluren’s CMA status in March 2025 (19). Following this decision, the Dutch CED program for ataluren was terminated in April 2025, and patients can no longer be treated with this medicine. Production of the second medicine, rhPTH 1–84 production ceased by the HTD in 2024. Following this, the CED program for rhPTH 1–84 ended, and patients currently using this medicine have been transitioned to a new treatment. Teduglutide is currently the only medicine in the CED program. Atidarsagene autotemcel is in the stage of drafting the process agreement and price negotiating (phase II). For the included medicines, the envisioned duration at the start of the conditional reimbursement was between 35 and 60 months.

### International comparison of reimbursement recommendations

To put the presented Dutch results into perspective, we aimed to understand how other HTA organizations approached the assessment of the products under investigation. Hence, Table 2 shows the reimbursement recommendations made by different HTA organizations for the medicines included in the Dutch CED program. Important to note is that this reflects only the publicly available recommendations made by HTA organizations and does not necessarily reflect the payment structures in place. Despite differences in national HTA methodologies and remit, other HTA organizations also often opted for risk-sharing measures. NICE recommended both OB-MEAs and FB-MEAs for the medicines involved. HAS often requires mandatory reassessments of their recommendation (while reimbursing the medicine in the meantime) and has recommended this for two of the six medicines. Additionally, HAS had two negative recommendations, one positive, and one OB-MEA recommendation. GBA did not recommend any MEAs, as it is not in their remit to do so, and came to three positive and two negative recommendations. CADTH did not have any recommendations for three of the six medicines and recommended FB-MEAs in the other cases.

**Table 2.** Initial HTA recommendations from other HTA organizations for the medicines in the CED program

INN	ZIN	NICE	HAS	GBA	CADTH
Entrectinib	OB-MEA	OB-MEA	Negative	Negative	FB-MEA
Larotrectinib	OB-MEA	OB-MEA	Mandatory reassessment	Negative	FB-MEA
Ataluren	OB-MEA	OB-MEA	Mandatory reassessment	Positive	NA
rhPTH 1–84	OB-MEA	NA	OB-MEA	NA	NA
atidarsagene autotemcel*	OB-MEA	FB-MEA	Negative	Positive	NA
teduglutide	OB-MEA	FB-MEA	Positive	Positive	FB-MEA

Abbreviations: HTA, health technology assessment; CED, Coverage with evidence development; ZIN, Zorginstituut Nederland; NICE, National Institute for Health and Care Excellence; HAS, Haute Autorité de Santé; GBA, Der Gemeinsame Bundesausschuss; CADTH, Canadian Agency for Drugs and Technologies in Health; FB-MEA, financial-based managed entry agreement; INN, International Nonproprietary Name; OB-MEA, outcome-based managed entry agreement; NA, recommendation not available. *The CED program is still under development for atidarsagene autotemcel.

## Discussion

The Dutch CED program aims to gather additional evidence on the clinical added benefit of OMPs, CMAs, and AECs with insufficient evidence regarding their added benefit while providing conditional patient access. Since 2019, six medicines have been included in the program or are current candidates (April 2025). The research proposal, process agreement, and financial agreement are tailor-made for individual products. The program is currently used to bridge evidence gaps in treatment populations, subgroups, or new types of registered indications. The comparison with HTA//reimbursement advice from other countries shows that other countries often also adopt additional coverage measures when assessing these medicines included in the Dutch CED program.

Uncertainty is an important challenge in assessing the value of a medicine, and it arises in almost all HTA reports (20). To deal with and share the risk of this uncertainty, HTA organizations often advise implementing MEAs. The Dutch CED program is a form of an OB-MEA. Earlier studies on CED programs from the United States, Switzerland, and the Netherlands found that they theoretically can be useful in obtaining earlier access to medicines or access to medicines with large uncertainty about their clinical benefit. However, there are also significant challenges in designing and implementing a CED program, such as the costs and efforts of setting up the required evidence generation, the duration of evidence gathering, the quality and type of data to collect, and reversing a coverage decision (11–14). An example illustrating these challenges was the “t = 0 t = 4 program” in the Netherlands. This conditional financing scheme was in effect between 2006 and 2012 and consisted of a CED program for expensive hospital medicines with a 4-year duration. This CED program focused on gathering data on appropriate use and cost-effectiveness in practice. A review of this program by Makady et al. found that although the program could theoretically provide an option for quick but conditional access, some aspects negatively affected its value in practice. The study found that of the 12 medicines that reached T = 4, 11 needed an extension and did not deliver the requested data within the envisioned 4 years. Secondly, the conducted outcome research during the 4 years did not provide sufficient evidence to inform decision-making at the time of reassessment (13;21). A positive aspect of the past CED program was the involvement of all stakeholders in the program (13;21). This program focused on the collection of RWE on appropriate use and cost-effectiveness, whereas the current CED program focuses on gathering clinical added benefit data. Despite this difference, important lessons have been incorporated into the program. In the new program, each CED agreement is specific to the medicine involved, the indication, and the research question that needs to be answered. Additionally, the current program opens up the possibility of having shorter schemes (as seen in the case studies) or longer schemes of up to 7 years or even 14 years in special instances (as proposed in the case of atidarsagene autotemcel). The involvement of all stakeholders has been intensified in the new program, as all parties are now involved in the drafting and signing of a process agreement.

So far, only a limited number of medicines have been included in the CED program. At the start of the program in 2019, it was foreseen that two medicines per year would enter the program. The reasons why this annual target was not met are not completely known. In an evaluation of the CED program with all stakeholders, published in January 2025, this issue was discussed. In principle, HTDs need to apply for the CED program, but ZIN facilitates this process by having exploratory meetings with HTDs about their products to discuss whether a medicine would be a good candidate. Over the last years, ZIN unsuccessfully invited the HTDs of four CMA products to apply for a CED program. This shows that several CMAs that would fit in the CED program are not included, hampering potential timely access for Dutch patients. A reason for this, as mentioned by an HTD, is that the time and money that need to be invested to set up data collection for Dutch patients as part of the CED are not worth the effort, and HTDs would rather wait for the evidence that is generated through the studies required by the EMA for their CMA status. Another point raised in the evaluation is that there are medicines that are not a CMA, AEC, or OMP that would benefit from the CED program. This suggests that the selection criterion regarding the CMA, AEC, or OMP status could be loosened. The MOH, in collaboration with ZIN, is currently researching whether this could be modified. Modification of the other selection criteria is not desired. Furthermore, the DAP and the Orphan Drug Access Protocol (ODAP) of the Dutch health insurers might also play a role. The DAP and the ODAP are alternative OB-MEAs that were implemented on a payer level that collect RWD for medicines with uncertain clinical benefit that are outside of the scope of ZIN and are often implemented earlier in their drug life cycle (18;22). Another critical point is that it is too soon to say how well this gathered clinical benefit data can answer the research gaps, as none of the drugs have gone through the full cycle, including the reassessment phase. Reasons for that include that two of the drugs (ataluren and rhPTH 1–84) did not reach the final phase of the CED program (reassessment) due to issues linked to their marketing authorization status: rhPTH 1–84 has been taken off the market by the HTD, and ataluren has lost marketing authorization, after multiple negative CHMP advices, in March 2025 because evidence requested by the EMA showed too little effectiveness. After the first negative CHMP advice in September 2023, no new patients were included in the Dutch CED program, but current patients were allowed to keep using the medicine, as the results on the specific subgroup in the Dutch CED program were still under debate, and the CHMP was still reassessing new data. After the negative EC decision, all current treatments in the CED program were also terminated. Currently, only teduglutide has been in the CED program since April 2025, and AA has not yet entered the data collection phase.

Still, preliminary findings from our case studies showed that the program can be used to gather additional clinical benefit data for OMPs, CMAs, and AECs that do not yet have enough evidence to be regularly reimbursed in the Dutch healthcare system, while at the same time providing controlled access. In conclusion, three important factors were identified that facilitate the process of the CED program: The first factor is the case-by-case evidence generation requirements and duration, followed by the thorough assessment of the feasibility of the research proposal by ZIN in phase I. The second is incorporating all stakeholders in drafting the process agreement in phase II. The last is the only-in-research approach during the coverage period. These findings add to the current literature by Walker et al. and Callenbach et al. that stress that outcome-based MEAs, such as CED, can be used to share the financial risk, gather additional data, and facilitate access to innovative therapies with uncertain (clinical) benefits. However, implementing these outcome-based MEAs should be done well-informed and on a case-by-case basis (9;23).

Disinvestment of medicines included in the CED program could be initiated due to either external actors, the HTD (for rhPTH 1–84), EMA (for ataluren), or a negative reimbursement advice in the regular ZIN assessment following the coverage period in the CED. To ensure an effective ending of the conditional situation, specific agreements regarding this process have been incorporated into the process agreement signed by all stakeholders. Disinvestment is a difficult process even in the presence of strong evidence that a medicine is (cost)ineffective (24). Only a few countries have disinvestment initiatives for healthcare in place, and only limited frameworks exist within HTA bodies on this subject (25;26). An evaluation study of disinvestment in Dutch healthcare showed that an active disinvestment process was more likely successful when there is sufficient support from stakeholders (e.g., clinicians, policymakers, and patient groups); it is possible to ease the effect of the disinvestment for patients, stakeholders themselves do not have a financial interest in maintaining reimbursement, and stakeholders are not inclined to exert pressure against disinvestment beyond their formal role (27). By including all stakeholders in drafting the process agreement, lessons from the evaluation study described above are incorporated as much as possible, but challenges will remain.

This Dutch CED program could be seen as an attempt to implement (a part of) life-cycle HTA. Life-cycle HTA aims to establish a cyclic process for HTA that is used to improve system sustainability, facilitate evidence generation, and lower decision-making uncertainty (28). An important aspect of life-cycle HTA is using RWE in HTA and reimbursement decision-making. RWE can, for instance, be used to add to the results from (single-arm) clinical trials or reflect on the long-term effectiveness of medicines (29). The use of RWE in HTA is challenging because of concerns regarding confounding by indication, data quality, data availability, and best practices (30;31). In the CED program, RWD from registries is used in addition to data gathered in clinical trials. When no more clinical trials are running or gathering the requested data, RWD from registries can be the only additional evidence gathered, showing the importance of multistakeholder registries (32). In the future, supranational initiatives on the topic of RWD, such as DARWIN-EU and the European Health Data Space, might provide additional sources of RWD and methods for RWD implementation in HTA (33;34). This could help international collaboration between HTA organizations in the future. Collaboration on the collection of RWD and the implementation of MEAs with similar outcome variables to be collected could increase the usefulness of the RWD collected and further decrease uncertainty in HTA.

A general limitation of this study is that the CED program has only been around for 5 years and no medicines have completed the regular CED program and have been reassessed. Therefore, we cannot yet study whether the collected data helps bridge the evidence gap. Furthermore, we noticed that compared with the growing number of OMPs, CMA, and AEC, only a few medicines were included in the CED program. We did not study why HTD did not opt to apply for the CED program further than has been done in a process evaluation by ZIN. Therefore, we do not have full information on other medicines that might have fit in the program, or that do not fit but might have been preferred to be included in a CED program by stakeholders, including ZIN. Future process evaluations, performed by ZIN and the MOH, should critically reflect whether the CED program truly helps bridge the evidence gaps and how to improve the number of medicines that enter the CED program.

## Conclusion

The Dutch CED program that started in 2019 gathers additional clinical data and facilitates access to OMPs, CMAs, and AECs that have insufficient evidence for regular reimbursement. Until now, six medicines with different types of evidence gaps have been included or are candidates in the program. Important facilitating factors of the current program are the involvement of all stakeholders, the “only-in-research” approach of data gathering, and the case-by-case evidence generation requirements and duration. However, the program does not yet include the expected number of medicines, and no conclusion can be drawn so far on the usefulness of the data collection, as there has not yet been a reassessment in the regular CED program until today. Continuous evaluation is needed to generate insights about the value of this program.

## References

[1] Orphan designation: Overview | European Medicines Agency. Available from: https://www.ema.europa.eu/en/human-regulatory-overview/orphan-designation-overview.

[2] Conditional marketing authorisation | European Medicines Agency. Available from: https://www.ema.europa.eu/en/human-regulatory-overview/marketing-authorisation/conditional-marketing-authorisation.

[3] Bouwman L, Sepodes B, Leufkens H, Torre C. Trends in orphan medicinal products approvals in the European Union between 2010–2022. Orphanet J Rare Dis. 2024;19(1):91. doi:10.1186/s13023-024-03095-z. 38413985 PMC10900541

[4] Manellari S, Musazzi UM, Rocco P, Minghetti P. Marketing authorisations for unmet medical needs: a critical appraisal of regulatory pathways in the European Union. Int J Pharm. 2023;642:123193. doi:10.1016/j.ijpharm.2023.123193. 37394157

[5] Mills M, Kanavos P. How do HTA agencies perceive conditional approval of medicines? Evidence from England, Scotland, France and Canada. Health Policy. 2022;126(11):1130–1143. doi:10.1016/j.healthpol.2022.08.005. 36050193

[6] Pinilla-Dominguez P, Naci H, Osipenko L, Mossialos E. NICE’S evaluations of medicines authorized by EMA with conditional marketing authorization or under exceptional circumstances. Int J Technol Assess Health Care. 2020;36(4):426–433. doi:10.1017/S0266462320000355. 32638664

[7] Vokinger KN, Kesselheim AS, Glaus CEG, Hwang TJ. Therapeutic value of drugs granted accelerated approval or conditional marketing authorization in the US and Europe from 2007 to 2021. JAMA Health Forum. 2022;3(8):e222685. doi:10.1001/jamahealthforum.2022.2685. 36200635 PMC9391955

[8] Callenbach MHE, Vreman RA, Mantel-Teeuwisse AK, Goettsch WG. When reality does not meet expectations—Experiences and perceived attitudes of Dutch stakeholders regarding payment and reimbursement models for high-priced hospital drugs. Int J Environ Res Public Health. 2023;20(1):340. doi:10.3390/ijerph20010340. PMC9819658 36612665

[9] Callenbach MHE, Goettsch WG, Mantel-Teeuwisse AK, Trusheim M. Creating win-win-win situations with managed entry agreements? Prioritizing gene and cell therapies within the window of opportunity. Drug Discov Today. 2024;29(7):104048. doi:10.1016/j.drudis.2024.104048. 38830504

[10] Performance-based managed entry agreements for new medicines in OECD countries and EU member states: How they work and possible improvements going forward. 2019;115. doi:10.1787/6e5e4c0f-en.

[11] Trueman P, Grainger DL, Downs KE. Coverage with evidence development: applications and issues. Int J Technol Assess Health Care. 2010;26(1):79–85. doi:10.1017/S0266462309990882. 20059784

[12] Brügger U, Horisberger B, Ruckstuhl A, Plessow R, Eichler K, Gratwohl A. Health technology assessment in Switzerland: a descriptive analysis of “coverage with evidence development” decisions from 1996 to 2013. BMJ Open. 2015;5(3):e007021. doi:10.1136/bmjopen-2014-007021. PMC4386218 25818273

[13] Makady A, van Veelen A, de Boer A, Hillege H, Klungel OH, Goettsch W. Implementing managed entry agreements in practice: the Dutch reality check. Health Policy. 2019;123(3):267–274. doi:10.1016/j.healthpol.2018.09.016. 30316540

[14] Zeitler E, Gilstrap L, Coylewright M, Slotwiner D, Carrie H., Colla P, Al-Khatib S. Coverage with evidence development: Where are we now? 2022;28. Available from: https://www.ajmc.com/view/coverage-with-evidence-development-where-are-we-now-. 10.37765/ajmc.2022.88870 35981123

[15] Ministerie van Volksgezondheid W en S. Conditional inclusion of orphan drugs, conditionals and exceptionals – About us – National Health Care Institute. February 14, 2020. Available from: https://english.zorginstituutnederland.nl/about-us/working-methods-and-procedures/conditional-inclusion-of-orphan-drugs-conditionals-and-exceptionals.

[16] Ministerie van Volksgezondheid W en S. Advising on and clarifying the contents of the standard health care benefit package – About us – National Health Care Institute. January 1, 2019. Available from: https://english.zorginstituutnederland.nl/about-us/working-methods-and-procedures/advising-on-and-clarifying-the-contents-of-the-standard-health-care-benefit-package.

[17] Ministerie van Volksgezondheid W en S. Assessment of outpatient medicines for the benefit of the Medicine Reimbursement System (GVS) – About us – National Health Care Institute. June 19, 2019. Available from: https://english.zorginstituutnederland.nl/about-us/working-methods-and-procedures/assessment-of-outpatient-medicines-for-the-benefit-of-the-medicine-reimbursement-system-gvs.

[18] Zeverijn LJ, van Doorn-Khosrovani van W SB, van Roy AAMGP, et al. Harmonising patient-access programmes: the Dutch DRUG access protocol platform. Lancet Oncol. 2022;23(2):198–201. doi:10.1016/S1470-2045(21)00707-5. 35114116

[19] Translarna | European medicines agency (EMA) . July 23, 2018. Available from: https://www.ema.europa.eu/en/medicines/human/EPAR/translarna.

[20] Vreman RA, Naci H, Goettsch WG, et al. Decision making under uncertainty: comparing regulatory and health technology assessment reviews of medicines in the United States and Europe. Clin Pharmacol Ther. 2020;108(2):350–357. doi:10.1002/cpt.1835. 32236959 PMC7484915

[21] Makady A, van Acker S, Nijmeijer H, et al. Conditional financing of drugs in the Netherlands: past, present, and future—results from stakeholder interviews. Value Health. 2019;22(4):399–407. doi:10.1016/j.jval.2018.11.016. 30975390

[22] Deesker LJ, Franssen CFM, Dorresteijn E, et al. Controlled access to lumasiran in primary hyperoxaluria type 1: evaluation of a new access route for orphan drugs in the Netherlands. Nephrol Dial Transpl. 2025;40:1887–1896. doi:10.1093/ndt/gfaf060. PMC12477465 40121182

[23] Walker S, Sculpher M, Claxton K, Palmer S. Coverage with evidence development, only in research, risk sharing, or patient access scheme? A framework for coverage decisions. Value Health. 2012;15(3):570–579. doi:10.1016/j.jval.2011.12.013. 22583469

[24] MacKean G, Noseworthy T, Elshaug AG, et al. Health technology reassessment: the art of the possible. Int J Technol Assess Health Care. 2013;29(4):418–423. doi:10.1017/S0266462313000494. 24290335 PMC3846380

[25] Calabrò GE, La Torre G, de Waure C, et al. Disinvestment in healthcare: an overview of HTA agencies and organizations activities at European level. BMC Health Serv Res. 2018;18(1):148. doi:10.1186/s12913-018-2941-0. 29490647 PMC5831213

[26] Kamaruzaman HF, Grieve E, Wu O. Disinvestment in healthcare: a scoping review of systematic reviews. Int J Technol Assess Health Care. 2022;38(1):e69. doi:10.1017/S0266462322000514. 35853843

[27] Rotteveel AH, Lambooij MS, van de Rijt JJA, van Exel J, Moons KGM, de Wit GA. What influences the outcome of active disinvestment processes in healthcare? A qualitative interview study on five recent cases of active disinvestment. BMC Health Serv Res. 2021;21(1):298. doi:10.1186/s12913-021-06298-3. 33794869 PMC8017606

[28] Trowman R, Migliore A, Ollendorf DA. Health technology assessment 2025 and beyond: lifecycle approaches to promote engagement and efficiency in health technology assessment. Int J Technol Assess Health Care. 2023;39(1). doi:10.1017/S0266462323000090. PMC11574536 36815310

[29] Bharmal M, Katsoulis I, Chang J, et al. Can real-world evidence (RWE) drive European, UK, and US payer decisions in the reassessment of oncology therapies? Value Health. 2022;25(12):S9. doi:10.1016/j.jval.2022.09.048.

[30] Graili P, Guertin JR, Chan KKW, Tadrous M. Integration of real-world evidence from different data sources in health technology assessment. J Pharm Pharm Sci. 2023;26:11460. doi:10.3389/jpps.2023.11460. 37529633 PMC10387532

[31] Claire R, Elvidge J, Hanif S, et al. Advancing the use of real world evidence in health technology assessment: insights from a multi-stakeholder workshop. Front Pharmacol. 2024;14:1289365. doi:10.3389/fphar.2023.1289365. 38283835 PMC10811058

[32] Schoenmakers DH, van den Berg S, Timmers L, et al. Framework for multistakeholder patient registries in the field of rare diseases. Neurology. 2024;103(6):e209743. doi:10.1212/WNL.0000000000209743. 39173102 PMC11379353

[33] **Data analysis and real world interrogation network (DARWIN EU) | European Medicines Agency (EMA)**. June 4, 2021. Available from: https://www.ema.europa.eu/en/about-us/how-we-work/data-regulation-big-data-other-sources/real-world-evidence/data-analysis-real-world-interrogation-network-darwin-eu.

[34] The European Health Data Space (EHDS). Available from: https://www.european-health-data-space.com/.

